# The association between intimate partner violence, psychiatric morbidity amongst pregnant women and partner alcohol use in southern Nigeria

**DOI:** 10.4102/phcfm.v12i1.2226

**Published:** 2020-07-02

**Authors:** Oluyemisi Adebowale, Bawo James

**Affiliations:** 1 Department of Clinical Services, Federal Neuro-Psychiatric Hospital, Benin City, Edo State, Nigeria

**Keywords:** intimate partner violence, psychiatric morbidity, pregnancy, partner alcohol use, Nigeria

## Abstract

**Background:**

Intimate partner violence (IPV) against women is common, although prevalence and correlates amongst pregnant women in developing countries are poorly researched.

**Aim:**

To identify the magnitude of IPV, and its relationship with psychiatric morbidity and partner alcohol use.

**Setting:**

This study was conducted among women receiving routine ante-natal care at a secondary level healthcare facility in southern Nigeria.

**Methods:**

A cross-sectional descriptive study was conducted by recruiting pregnant women (*n* = 395) attending the Ante-Natal Clinic of the Central Hospital, Benin-City, Edo State, Nigeria, between August 2015 and February 2016 and undertaking face-to-face interviews utilising a socio-demographic questionnaire, the Composite Abuse Scale and the 20-item Self-reporting Questionnaire.

**Results:**

Past 12-month prevalence of IPV was 24.8%, with emotional abuse being the commonest type (89.8%). Forty-six participants (11.6%) screened positive for probable psychiatric morbidity. Predictors of IPV included partner alcohol use in the past 12 months (adjusted odds ratio [aOR]: 2.67; 95% confidence interval [CI]: 1.16–6.16; *p* < 0.02), having a psychiatric morbidity (aOR: 2.53; 95% CI: 1.27–5.04; *p* < 0.01), being single (aOR: 2.12; 95% CI: 1.25–3.58; *p* < 0.01) and multiparous (aOR: 2.5; 95% CI: 1.43–4.38; *p* < 0.001).

**Conclusion:**

Intimate partner violence was common amongst pregnant women in Nigeria. Identified modifiable risk factors can be targets for screening and intervention for women in these settings.

## Introduction

In the past decade, increasing research has focused on violence perpetuated on women by their intimate partners. Most research indicates that women are more likely to be victimised by almost every type of intimate partner violence (IPV).^1^ Intimate partner violence is defined as actual or threatened physical, sexual, psychological or stalking violence by current or former intimate partners.^1^ Overall, at least one in three of the world’s female population has been physically or sexually abused by a partner at some time in their life. Furthermore, physical violence is almost always accompanied by emotional abuse and in many cases by sexual abuse.^2^

Domestic violence has been reported to occur even during pregnancy^3,4^ with overlapping variables at group and personal levels. Globally, one in every four women is physically or sexually abused during pregnancy, usually by her partner.^2^ Surprisingly, there is a dearth of national data on the subject of IPV amongst women, although available data suggest that violence against the Nigerian woman is common, yet, very few studies have been conducted for pregnant women.^5,6,7,8^ Experiences of IPV have long-term negative physical and mental health consequences for victims, even after the abuse has ended.^9,10^ Prevalence of anxiety and depression is common amongst the victims of IPV when compared with women who did not face IPV.^11,12^ Researchers have noted a strong relationship between IPV prior to or during pregnancy and mental health disorders.^13,14^ These can result in a poor quality of life and high utilisation of health services.^15,16,17,18^ Alcohol abuse by partners has been linked with acts of violence.^15^

The aim of this study was to examine the magnitude and characteristics of IPV as well as to assess the extent to which violence by intimate partners is associated with psychiatric morbidity and partners’ alcohol use.

## Methods

### Study setting

This cross-sectional descriptive study was conducted between August 2015 and February 2016 at the Ante-Natal Clinics of the Central Hospital, Benin-City, Edo State, Nigeria, which is the largest secondary health care facility in the city. It provides services to the communities comprising the three local government areas that make up Benin-City, with an approximate population of 1 million.

### Study sample and procedure

The sample size was calculated by using the formula for proportions^19^ with a 95% confidence interval (CI) and an error margin of 0.05. Three hundred and ninety-five pregnant women aged between 16 and 49 years attending the follow-up clinic were recruited after they gave informed written consent (assent for those less than 18 years). Those who declined participation or could not communicate in the English language were excluded and were replaced by the next woman on the clinic list (sample frame). A systematic random sampling technique was employed in the selection of study participants. Interviews were conducted in a private consulting room to ensure safety and confidentiality and minimise distress to participants. Questionnaires were administered during face-to-face interviews, and information on adequate referral and support was provided to those who needed it.

## Instruments

### Socio-demographic questionnaire

A semi-structured socio-demographic questionnaire was designed by the authors to elicit information on the following variables: age, marital status, educational level, parity and employment status. Participants’ reports of partner lifetime and 12-month alcohol use were also obtained.

### Composite Abuse Scale

The Composite Abuse Scale (CAS) is a 30-item validated research instrument that is based on the concept of IPV that includes not only violent acts but also coercion arising out of conflict. It is recommended as an IPV research assessment tool by the National Centre for Injury Prevention and Control^20^ because it has demonstrated a high level of reliability and validity in studies exploring self-reported prevalence of IPV. It has a reliability score (Cronbach’s alpha) of 0.90 or more for each subscale and an all-item total correlation score of 0.6 or above.^21^ A cut-off score of 7 was adopted for this study to divide participants into ‘abused’ and ‘non-abused’ categories.^21^ Subscales of physical abuse, emotional abuse, harassment and severe combined abuse with cut-offs of 2, 4, 2 and 1 respectively determined the pattern of abuse amongst those reported IPV.^21^ For this study, the CAS was reviewed by a female psychiatrist, a female social health worker and a male clinical psychologist for face validity, and the outcome was satisfactory. The CAS had a good reliability score (Cronbach’s alpha) of 0.92 in this cohort.

### Self-reporting Questionnaire-20

The Self-reporting Questionnaire-20 (SRQ-20) was developed as part of a collaborative study coordinated by the World Health Organization (WHO)^22^ on strategies for extending mental health care. It consists of 20 yes or no questions with a reference period to the previous 30 days. It has acceptable levels of reliability and validity in many settings and is recommended by the WHO as a screening tool for psychiatric morbidity. It has previously been used to screen for maternal illness in developing countries (including Ethiopia) of similar socio-cultural setting^23^ and a cut-off score of 7/8 was used to separate probable non-cases from cases of common mental disorder.

The scale consists of 20 dichotomous items covering depression, anxiety and somatisation symptoms. Scores range from 0 to 20 and implicitly increase with the degree of psychological distress. The SRQ was validated in a primary care setting in rural south-western Nigeria^24^ and found to effectively discriminate between patients with and without psychiatric morbidity. This was best performed at a cut-off point of 5, which has the optimal sensitivity of 98.8% and specificity of 90.9%.^24^ A cut-off point of 5 was therefore used for this study.

## Data analysis

The data collected were analysed by using the Statistical Package for the Social Sciences version 20.^25^ The dependent variable was the presence or absence of IPV on the CAS-20, which was compared against socio-demographic characteristics, the presence of psychiatric morbidity and partner alcohol use. The chi-square test was used to analyse categorical variables and test proposed hypotheses. A binary logistic regression analysis was performed to determine the predictors of IPV. For all analyses, the level of statistical significance was set at *p* ≤ 0.05 *a priori*.

### Ethical consideration

Ethical clearance was obtained from the Ethics Committee of the Federal Neuro-Psychiatric Hospital, Uselu, Benin City (Ref: T/A.740/44), and the Edo State Ministry of Health prior to commencement of the study. Those with Self-reporting Questionnaire-20 (SRQ-20) scores indicative of mental ill health received counselling and a referral to a mental health practitioner. Victims of IPV were counselled about seeking help.

## Results

A total of 412 pregnant women were approached for consent to participate in this study. Three hundred and ninety-five (395) gave consent and were recruited giving a response rate of 95.87%.

### Socio-demographic characteristics

Participants were aged between 16 and 44 years with a mean age (standard deviation [s.d.]) of 30.05 (5.3) years. About a quarter (*n* = 84; 21.3%) were unemployed and amongst those employed, most were service and sales workers (178/311; 57.2%). Two hundred and seventy-two (68.8%) participants were married, and 115 (29.1%), (*n* = 145; 36%) were multiparous (Table 1).

**TABLE 1:** Socio-demographic characteristics of participants (*N* = 395).

Variable	Total	
	*n*	%
**Age class**		
16–26	100	25.3
27–37	260	65.8
38–49	35	8.9
**Ethnic group**		
Benin	199	50.4
Esan	61	15.4
Delta Igbo	17	4.3
Igbo	23	5.8
Etsako	24	6.1
Others	71	18.0
**Religion**		
Christian	382	96.7
Muslim	13	3.3
**Level of education**		
Nil formal education	7	1.8
Primary	45	11.4
Secondary	198	50.1
Tertiary	141	36.7
**Employment status**		
Employed	311	78.7
Unemployed	84	21.3
**Occupation (ISCO-08)† (*n* = 311)**		
Managers	4	1.3
Professionals	43	13.8
Technicians and associate professionals	5	1.6
Clerical support workers	14	4.5
Service and sales workers	178	57.2
Craft and related trade workers	65	20.9
Elementary occupation	2	0.6
**Parity**		
Nulliparous	142	36.0
Primiparous	102	25.8
Multiparous	145	36.7
Grand-multiparous	6	1.5

† , ISCO-08, International standard classification of occupation, 2008.

### Prevalence and patterns of intimate partner violence

**FIGURE 1:**
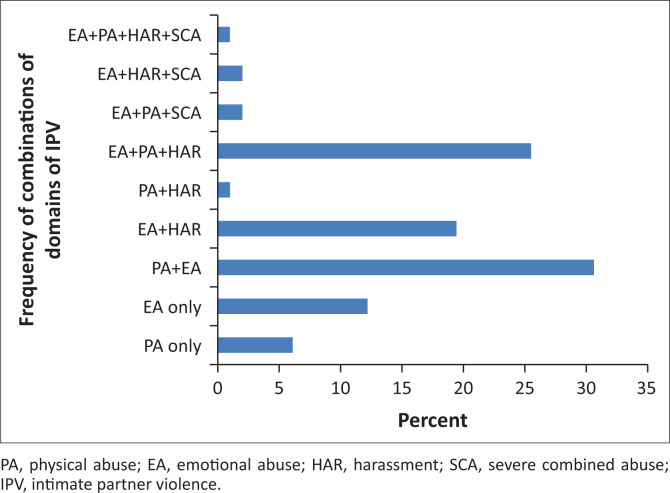
Pattern of intimate partner violence experienced by victims.

Ninety-eight (24.8%) participants reported experiencing IPV in the past year. Emotional abuse was most commonly reported, occurring singly and in combination with other forms in 89.8% of those who suffered IPV. The severe combined abuse domain of the CAS (which assesses for sexual abuse) was the least reported (5/98, 5.1%), occurring only in combination with other domains. The majority of participants who suffered IPV reported experiences of a combination of physical and emotional violence suffered at the hands of their partners (*n* = 30, 30.6%) (Table 2) (Figure 1).

**TABLE 2:** Prevalence of intimate partner violence amongst participants (*N* = 395).

Variable	Total	
	*n*	%
**Intimate partner violence**		
Present	98	24.8
Absent	297	75.2
**Domains of IPV on the CAS experienced by victims in combinations (*n* = 98)**		
Physical abuse	66/98	67.3
Emotional abuse	88/98	89.8
Harassment	47/98	48.0
Severe combined abuse	5/98	5.1

IPV, intimate partner violence; CAS, Composite Abuse Scale.

### Socio-demographic correlates of intimate partner violence

Women who were single (*p* < 0.001) and those with at least a previous birth (*p* < 0.01) were significantly more likely to experience IPV; however, no associations were observed with age (*p* = 0.89), educational status (*p* = 0.149) and employment status (*p* = 0.459) (Table 3).

**TABLE 3:** Association between socio-demographic characteristics of participants and presence of intimate partner violence.

Variable	IPV		Statistic	
	Present (*n*)	Absent (%)	*X*^2^ (df = 1)	*p*
**Age distribution**				
≤ 30 years	53	163	0.019	0.890
> 30 years	45	134	-	
**Educational status**				
> 12 years of formal education	30	115	2.085	0.149
≤ 12 years of formal education	68	182	-	
**Employment status**				
Employed	80	232	0.550	0.459
Unemployed	18	65	-	
**Occupational status**				
High	11	55	3.374	0.066
Low	68	177	-	
**Marital status**				
Single	44	79	11.506	0.001
Currently married	54	218	-	
**Parity**				
Primi-, multi- and grand multiparous	74	179	7.433	0.006
Nulliparous	24	118	-	

IPV, intimate partner violence; *X*^2^, chi-square; df, degree of freedom.

### Psychiatric morbidity and intimate partner violence

Forty-six (11.6%) participants screened positive for psychiatric morbidity. Those who experienced IPV in the preceding 12 months were nearly four times more likely to have a psychiatric morbidity compared with those who did not experience IPV (crude odds ratio [OR]: 3.65, 95% CI: 1.84–7.21, *p* < 0.001). Pregnant women reporting physical abuse (*p* < 0.001), emotional abuse (*p* < 0.001) and harassment (*p* < 0.03) were significantly more likely to screen positive for a psychiatric morbidity. Those with experiences of emotional violence had the highest likelihood for a probable psychiatric morbidity (crude OR: 3.54, 95% CI: 1.87–6.69, *p* = 0.001) (Table 4).

**TABLE 4:** The association between psychiatric morbidity and various domains of intimate partner violence.

Domains of IPV	SRQ-20: *N* = 395		Statistics			
	Positive (*n*)	Negative (%)	Crude OR	95% CI	*X*^2^ (df = 1)	*p*
**Physical abuse**						
Present	16	50	3.19	1.62–6.27	12.220	0.001*
Absent	30	299	-	-	-	
**Emotional abuse**						
Present	21	67	3.54	1.87–6.69	16.427	0.001*
Absent	25	282	-	-	-	
**Harassment**						
Present	10	37	2.34	1.08–5.11	4.809	0.03*
Absent	36	212	-	-	-	
**Severe combined abuse**						
Present	2	3	-	-	1.658†	0.198
Absent	44	346	-	-	-	

IPV, intimate partner violence; SRQ-20, Self-Reporting Questionnaire-20; *X*^2^, chi-square; df, degree of freedom; Crude OR, Crude odds ratio, 95% CI, 95% confidence interval.

† , Yates corrected.

* , Significant values.

### Partner alcohol use and intimate partner violence

One hundred and seventy-eight (45.1%) women reported that their partner had used alcohol in the preceding 12 months, and 225 (57%) reported a lifetime history of alcohol use amongst their partners. Pregnant women who reported that their partners used alcohol in the preceding 12 months were three times more likely to report IPV (Crude OR: 3.41, 95% CI: 2.05–5.71, *p* < 0.001), and those whose partners had a lifetime alcohol use were four times more likely to report IPV (Crude OR: 4.3, 95% CI:2.43–7.88, *p* < 0.001) See Table 5.

**TABLE 5:** The association between partners’ alcohol use and intimate partner violence.

Partners’ alcohol use	IPV				Statistic			
	Present		Absent		Crude OR	95% CI	*X*^2^	*p*
	*n*	%	*n*	%				
**12 months**								
Yes	66	37.08	112	62.92	3.41	2.05-5.71	26.14	0.001*
No	32	14.75	185	85.25	-	-	-	
**Lifetime**								
Yes	79	35.11	146	64.89	4.3	2.43-7.88	29.74	0.001*
No	19	11.18	151	88.82	-	-	-	

IPV, intimate partner violence; *X*^2^, chi-square; Crude OR, Crude odds ratio; 95% CI, 95% confidence interval.

* , Significant values.

### Predictors of intimate partner violence

Following a binary logistic regression, the predictors of IPV included partner alcohol use in the past 12 months (adjusted OR: 2.67; 95% CI: 1.16–6.16; *p* < 0.02), having a psychiatric morbidity (adjusted OR: 2.53; 95% CI: 1.27–5.04; *p* < 0.01), being single (adjusted OR: 2.12; 95% CI: 1.25–3.58; *p* < 0.01) and multiparous (adjusted OR: 2.5; 95% CI: 1.43–4.38; *p* < 0.001) (Table 6).

**TABLE 6:** Predictors of intimate partner violence.

Variables	*B*	SE	Wald	df	OR	95% CI	*p*
Nulliparous	−0.927	0.287	10.418	1	0.4	0.23–0.70	0.001*
Not married	0.751	0.268	7.866	1	2.12	1.25–3.58	0.005*
Psychiatric morbidity present	0.929	0.351	7.002	1	2.53	1.27–5.04	0.008*
Partner alcohol use (12 month)	0.982	0.427	5.291	1	2.67	1.16–6.16	**0.**021*
Partner alcohol use (lifetime)	0.418	0.380	1.208	1	1.52	0.72–3.20	**0.272**

*B*, Regression coefficient; SE, standard error of regression coefficient; Wald, Wald chi-square; df, degree of freedom; OR, odds ratio; 95% CI, 95% confidence interval.

* , Significant values.

## Discussion

Based on the results of this study it could be noted that a quarter of the women reported IPV within the past year. This rate is consistent with previous research.^11,26,27,28^ Gyuse et al.^29^ and Fawole et al.^30^ however reported a lower prevalence of 11.1% amongst respondents in their current pregnancy and 14.2% in the preceding 12 months in Nigeria. The lower prevalence reported in these studies may be because of methodological differences. For instance, Fawole et al.^30^ reported that they excluded women who expressed fear that if they participated in the study and participated in the interview further violence may ensue, leading to a sampling bias. Also, the study used a self-designed questionnaire with a few semi-structured questions. Gyuse et al. also reported that ‘majority of participants not being able to identify a timing pattern for the abuse’.^29^ This may have resulted in an underestimation of the true prevalence. Higher prevalence of 31% has been reported by Hoque et al. in South-Africa amongst respondents in their current pregnancy.^31^ This brings to light the fact that experiences of violence during pregnancy are high in Africa.

Emotional abuse was the most commonly reported pattern of abuse occurring in 89.8% of participants reported IPV and is consistent with other studies from Nigeria,^11,29,32^ and other parts of Africa.^27,28,31^ The preponderance of emotional abuse may reflect the perception of partners that emotional abuse is least offensive and more tolerable by their spouse or the community in which they live.^32^ Additionally, most cultures in Nigeria place the male as the ‘father’ of the household,^33^ as such, wives are not spared of insults when being reprimanded. Paradoxically, some women express approval of emotional abuse from partners when home chores are not completed.^33^ In African settings, emotional abuse, harassment and sexual abuse are sometimes trivialised.^8,33^ As such, past research and advocacy have focused majorly on physical abuse, neglecting other forms of abuse.^32,34,35^ Public education emphasising the harmful effects of all forms of IPV with more emphasis on emotional violence and targeted especially at the male population may help reduce its occurrence.

Questions may also arise regarding the validity of the ‘emotional abuse’ construct on the CAS in this cultural setting because what constitutes emotional abuse on the CAS may be an acceptable way of communication. The measurement of emotional violence across cultures is complex, and there is a relative scarcity of research on emotional abuse in comparison with studies on physical or sexual violence.^36^ Despite the importance that women place on this form of violence, to date, there has been little methodological work to explore the best means to elicit and measure such experiences. There is a need for further validation of the CAS beyond face validity in Nigeria.

Over two-thirds (67.3%) of participants reporting violence reported physical abuse by their partners, and contrasting results (4.9%) were reported by Onoh et al.^32^ Onoh et al.^32^ focused primarily on ‘beating’, but physical abuse is much broader than ‘beating’. The CAS comprehensively assesses for physical abuse and explains the higher prevalence in this study. Spanking is accepted in Nigeria as an appropriate method for correcting an erring child.^37^ The ‘fatherly’ role the traditional African male plays in the general family setting may explain his use of physical violence as a means of instilling discipline in his dealings with his partner.^37,38^

Severe combined abuse (which assesses for sexual violence on the CAS) was reported in 5 of 98 (5.1%) persons who reported IPV. A higher prevalence of sexual abuse has been reported from Africa.^27,28^ Participants whose partners used alcohol in the preceding 12 months or in their lifetime were more likely to report IPV. Perpetrators of IPV have reported that they were under the influence of alcohol during the act.^39^ Women have also reported they perceived that the use of alcohol and drugs by their partners influenced their experience of IPV.^40^ Established pathways linking alcohol abuse and IPV include raised levels of aggression, misinterpretation of verbal or non-verbal cues, increased risk-taking behaviour and the fact that alcohol usage might be a source of argument in relationships.^41^

In this study, a little over a 10th (11.6%) of participants screened positive for a psychiatric morbidity. This study showed significant associations between experiences of violence and probable psychiatric morbidity in participants. Age, education, religion, ethnic group or employment status or occupation did not show this association. A similar association of case-ness for a mental illness and the experience of partner violence has been reported.^42,43^ In more specific terms, women exposed to IPV have been reported to have a higher incidence and severity of depression and anxiety symptoms, Post Traumatic Stress Disorder (PTSD) and suicidal thoughts.^11,13,44^

Furthermore, except for severe combined abuse, emotional and physical abuses were significantly associated with probable psychiatric morbidity. On the contrary, it is possible that the presence of a psychiatric illness in an individual, for example depression, may influence interpretations of verbal and non-verbal cues of a partner. A negative view of self, environment and the future caused by an illness may lead to frequent misinterpretation of intents and contents of communication, hence, a possible over-reporting of emotional abuse.^45^ This may account for the increased risk for a probable psychiatric morbidity amongst those reporting emotional abuse. Because of the cross-sectional nature of this study, it is not possible to establish whether exposure to violence occurred before or after the onset of symptoms of psychiatric morbidity. Therefore, determining the temporal relationship between the experiences of IPV and indicators of a psychiatric morbidity can be difficult. Theoretically, women with psychiatric morbidity may be at risk of experiencing violence in their relationships.^46,47^ However, studies on women’s health, by use of longitudinal designs and theoretical reasoning, suggest that the reported mental health problems are mainly the outcomes of abuse.^14,43^ The exposure to violence from an intimate partner could thus be a precipitating and a perpetuating factor for a mental illness. It has been reported that experiences of physical or sexual violence, or both, by a partner are associated with increased odds of reports of poor mental health.^43^ This effect is found to be irrespective of where a woman resides, her cultural or racial background or the extent to which violence might be tolerated or accepted in her society or by herself.^43^

Physical symptoms such as tiredness, diarrhoea, chest pain, poor sleep, poor appetite and poor digestion have been found to be associated with IPV.^48^ These symptoms have been shown to have associations with anxiety, somatic and depressive disorders that the SRQ-20 screens for.^24^ This emphasises the need for medical practitioners in antenatal care settings and general practice to routinely ask questions about IPV. The ante-natal clinic is an appropriate venue where women could be educated about proper ways of addressing and reporting issues of IPV. Common symptoms and early warning signs of a mental illness could be incorporated into ante-natal health talks. This will facilitate quick referrals to the psychiatrists, ensure early treatment and reduce disability, thereby enhancing the overall quality of life of the Nigerian woman.

Lastly, we also confirm that partner alcohol use increased the odds of reporting IPV. Although it would have been ideal to quantify the severity of use, we agree that it would be difficult to delineate factors that predispose to partner alcohol use, and the alcohol use itself, as it relates to IPV. We suggest that future studies are urgently required to examine the severity, contributing factors and perpetuating factors for partner alcohol use to refine strategies that would ameliorate this modifiable risk factor’s contribution to IPV. Our findings should be interpreted with the following limitations. Firstly, the use of a secondary healthcare facility and the urban-based nature of the study may restrict the generalisation of our findings to the wider settings. Secondly, some of the questions in the CAS may not be culturally adapted to describe IPV from the Nigerian perspective. Although selected for its comprehensiveness and strong psychometric properties, it has not been extensively validated beyond face validity and reliability in Nigeria. Thirdly, questions about partners’ use of alcohol were based on the respondents’ knowledge rather than self-report, making these variables less reliable.

## Conclusion

Intimate partner violence is common amongst pregnant women presenting at the ante-natal clinic of Central Hospital, Benin-City. Most reported experiences of combinations of various forms of violence. Emotional abuse was most commonly reported amongst participants in abusive relationships. About one-tenth of participants screened tested positive for a psychiatric morbidity. There is a need to institute interventions in ante-natal settings that educate pregnant women about the nature, risks and complications of IPV as well as the provision of resources to aid coping and mitigation of IPV.

## References

[1] Thompson M, Basile K, Hertz M, Sitterle D Measuring intimate partner violence victimization and perpetration: A compendium of assessment tools [homepage on the Internet]. Atlanta, GA: Centers for Disease Control and Prevention, National Center for Injury Prevention and Control; 2006 [cited 2014 Sep 23]. Available from: http://scholar.google.com/scholar?hl=en&btnG=Search&q=intitle:No+Title#0

[2] Heise L, Ellsberg M Ending violence against women. WHO. 1999;XXVII(4):1–44.

[3] Escribà-Agüir V, Ruiz-Pérez I, Saurel-Cubizolles M-J Screening for domestic violence during pregnancy. J Psychosom Obstet Gynecol. 2007;28(3):133–134. 10.1080/01674820701293155 17577754

[4] Rietveld L, Lagro-Janssen T, Vierhout M, Wong SLF Prevalence of intimate partner violence at an out-patient clinic obstetrics gynecology in the Netherlands. J Psychosom Obstet Gynecol. 2010;31(1):3–9. 10.3109/01674820903556388 20121462

[5] Aimakhu CO, Olayemi O, Iwe C, et al Current causes and management of violence against women in Nigeria. J Inst Obstet Gynaecol [serial online]. 2004 [cited 2014 Sep 25];24(1):58–63. Available from: http://www.ncbi.nlm.nih.gov/pubmed/14675983 10.1080/01443610310001620314 14675983

[6] Aihie O Prevalence of domestic violence in Nigeria: Implications for counselling. Edo J Couns. 2010;2(1):1–8. 10.4314/ejc.v2i1.52648

[7] Okemgbo CN, Omideyi AK, Odimegwu CO Prevalence, patterns and correlates of domestic violence in selected Igbo communities of Imo State, Nigeria. Afr J Reprod Health [serial online]. 2002 [cited 2014 Sep 11];6(2):101–114. Available from: http://www.ncbi.nlm.nih.gov/pubmed/12476722 12476722

[8] Antai D, Antai J Collective violence and attitudes of women toward intimate partner violence: Evidence from the Niger Delta. BMC Int Health Hum Rights [serial online]. 2009 [cited 2016 Feb 12];9:12 Available from: http://www.pubmedcentral.nih.gov/articlerender.fcgi?artid=2702345&tool=pmcentrez&rendertype=abstract 10.1186/1472-698X-9-12 PMC2702345 19508708

[9] Stenson K, Heimer G, Lundh C, Nordström ML, Saarinen H, Wenker A The prevalence of violence investigated in a pregnant population in Sweden. J Psychosom Obstet Gynecol. 2001;22(4):189–197. 10.3109/01674820109049973 11840572

[10] Campbell JC Violence against women II Health consequences of intimate partner violence. Lancet. 2002;359:1331–1336. 10.1016/S0140-6736(02)08336-8 11965295

[11] Mapayi B, Makanjuola ROA, Mosaku SK, et al Impact of intimate partner violence on anxiety and depression amongst women in Ile-Ife, Nigeria. Arch Womens Ment Health. 2013;16(1):11–18. 10.1007/s00737-012-0307-x 22936117

[12] Naeem F, Irfan M, Zaidi Q, Kingdon D, Ayub M Angry wives, abusive husbands: Relationship between domestic violence and psychosocial variables. Women Health Iss. 2008;18(6):453–462. 10.1016/j.whi.2008.08.002 18926727

[13] Beydoun HA, Beydoun MA, Kaufman JS, Lo B, Zonderman AB Social Science & Medicine Intimate partner violence against adult women and its association with major depressive disorder, depressive symptoms and postpartum depression: A systematic review and meta-analysis. Soc Sci Med. 2012;75(6):959–975. 10.1016/j.socscimed.2012.04.025 22694991 PMC3537499

[14] Howard LM, Oram S, Galley H, Trevillion K, Feder G Domestic violence and perinatal mental disorders: A systematic review and meta-analysis. PLoS Med. 2013;10(5):e1001452 10.1371/journal.pmed.1001452 23723741 PMC3665851

[15] Andersson N, Omer K Male responsibility and maternal morbidity: A cross-sectional study in two Nigerian states. BMC Heal Serv [serial online]. 2011 [cited 2014 Sep 25]. Available from: http://www.biomedcentral.com/1472-6963/11/S2/S7/10.1186/1472-6963-11-S2-S7 PMC3332566 22375828

[16] Tollestrup K, Sklar D, Frost FJ, et al Health indicators and intimate partner violence among women who are members of a managed care organization. Prev Med (Baltim). 1999;29(5):431–440. 10.1006/pmed.1999.0552 10564635

[17] McCauley J, Kern DE, Kolodner K, et al The ‘battering syndrome’: Prevalence and clinical characteristics of domestic violence in primary care internal medicine practices. Ann Intern Med. 1995;123(10):737–746. 10.7326/0003-4819-123-10-199511150-00001 7574191

[18] Plichta SB Intimate partner violence and physical health consequences: Policy and practice implications. J Interpers Violence. 2004;19(11):1296–1323. 10.1177/0886260504269685 15534333

[19] Kish L Survey sampling. New York, NY: John Wiley & Sons Inc.; 1965.

[20] Thompson MP, Basile KC, Hertz MF, Sitterle D Measuring intimate partner violence victimization and perpetration: A compendium of assessment tools. Atlanta, GA: Centers for Disease Control and Prevention, National Center for Injury Prevention and Control, 2006; p. 121–150.

[21] Hegarty K, Bush R, Sheehan M The composite abuse scale: Further development and assessment of reliability and validity of a multidimensional partner abuse measure in clinical settings. Violence Vict. 2005;20(5):529–547. 10.1891/vivi.2005.20.5.529 16248489

[22] Beusenberg M, Orley J A user’s guide to the self reporting questionnaire (SRQ). Geneva: World Health Organization, 1994; p. 84.

[23] Harpham T, Huttly S, De Silva MJ, Abramsky T Maternal mental health and child nutritional status in four developing countries. J Epidemiol Community Health [serial online]. 2005 [cited 2016 Feb 22];59(12):1060–1064. Available from: http://www.pubmedcentral.nih.gov/articlerender.fcgi?artid=1732980&tool=pmcentrez&rendertype=abstract 10.1136/jech.2005.039180 PMC1732980 16286495

[24] Abiodun AO Sensitivity and validity of the SRQ-20 in a primary health care centre in a rural community in Nigeria. Psychopathol Afr. 1988;xxii(1):79–88.

[25] Armonk IBM SPSS statistics for Windows, Version 20.0. Armonk, NY: IBM Corp; 2011.

[26] Ezechi OC, Kalu BK, Ezechi LO, Nwokoro CA, Ndububa VI, Okeke GC Prevalence and pattern of domestic violence against pregnant Nigerian women. J Obstet Gynaecol (Lahore). 2004;24(6):652–656. 10.1080/01443610400007901 16147605

[27] Tariku L, Tefera B, Meseret T Prevalence and associated factors of intimate partner violence during pregnancy among recently delivered women in public health facilities of Hossana Town, Hadiya Zone, Southern Ethiopia. Open Access Libr J. 2014;1:1–9. 10.4236/oalib.1100997

[28] Dunkle K, Jewkes R, Brown H, Yoshihama M, Gray G Prevalence and patterns of gender-based violence and revictimization among women attending antenatal clinics in Soweto, South Africa. Am J Epidemiol. 2004;160(3):230–239. 10.1093/aje/kwh194 15257996

[29] Gyuse A, Ushie A Pattern of domestic violence among pregnant women in Jos, Nigeria. S Afr Fam Pract. 2009;51(4):343–345. 10.1080/20786204.2009.10873877

[30] Fawole A., Hunyinbo K., Fawole OI Prevalence of violence against pregnant women in Abeokuta, Nigeria. J Obstet Gynaecol. 2008;48(4):405–414. 10.1111/j.1479-828X.2008.00868.x 18837847

[31] Hoque ME, Hoque M, Kader SB Prevalence and experience of domestic violence among rural pregnant women in KwaZulu-Natal, South Africa. S Afr J Epidemiol Infect. 2009;24(4):34–37. 10.1080/10158782.2009.11441360

[32] Onoh R, Umeora O, Ezeonu P, Onyebuchi A, Lawani O, Agwu U Prevalence, pattern and consequences of intimate partner violence during pregnancy at Abakaliki South-east Nigeria. Ann Med Health Sci Res 2013;3(4):484–491. 24379996 10.4103/2141-9248.122048 PMC3868111

[33] Kunnuji MON Experience of domestic violence and acceptance of intimate partner violence among out-of-school adolescent girls in Iwaya Community, Lagos State. J Interpers Violence. 2015;30(4):543–564. 10.1177/0886260514535261 24919993

[34] Owoaje ET, Olaolorun FM Women at risk of physical intimate partner violence: A cross-sectional analysis of a low-income community in southwest Nigeria. Afr J Reprod Heal. 2012;16(1):43–53. 22783667

[35] Fawole OI, Aderonmu AL, Fawole AO Intimate partner abuse: Wife beating among civil servants in Ibadan, Nigeria. Afr J Reprod Health. 2005;9(2):54 10.2307/3583462 16485586

[36] Garcia-Moreno C, Jansen H, Ellsberg M, Heise L, Watts C Prevalence of intimate partner violence: Findings from the WHO multi-country study on women’s health and domestic violence. Lancet [serial online]. 2006 [cited 2014 Sep 26];368(9543):1260–1269. Available from: http://www.ncbi.nlm.nih.gov/pubmed/17027732 10.1016/S0140-6736(06)69523-8 17027732

[37] Antai D Controlling behavior, power relations within intimate relationships and intimate partner physical and sexual violence against women in Nigeria. BMC Public Health [serial online]. 2011 [cited 2014 Sep 20];11:511 Available from: http://www.pubmedcentral.nih.gov/articlerender.fcgi?artid=3161889&tool=pmcentrez&rendertype=abstract 10.1186/1471-2458-11-511 PMC3161889 21714854

[38] Antai D, Antai J Attitudes of women toward intimate partner violence: A study of rural women in Nigeria. Rural Remote Heal [serial online]. 2008 [cited 2014 Oct 15];8(996). Available from: http://www.rrh.org.au/articles/subviewnew.asp?articleid=996 18842071

[39] Brisibe S, Ordinioha B, Dienye PO Intersection between alcohol abuse and intimate partner’s violence in a rural Ijaw Community in Bayelsa State, South-South Nigeria. J Interpers Violence. 2012;27(3):513–522. 10.1177/0886260511421676 21987506

[40] Ilika AL, Okonkwo PI, Adogu P Intimate partner violence among women of childbearing age in a primary health care centre in Nigeria. Afr J Reprod Health. 2002;6(3):53 10.2307/3583257 12685409

[41] Gordis E Alcohol, violence and aggression. Rockville, MD: National Institute on Alcohol Abuse and Alcoholism, 1997; p. 38.

[42] Ola B, Crabb J, Tayo A, Gleadow Ware SH, Dhar A, Krishnadas R Factors associated with antenatal mental disorder in West Africa: A cross-sectional survey. BMC Pregnancy Childbirth [serial online]. 2011 [cited 2014 Sep 1];11(1):90 Available from: http://www.biomedcentral.com/1471-2393/11/90 10.1186/1471-2393-11-90 PMC3231953 22054304

[43] Garcia-Moreno C, Ellsberg M, Jansen HA, Heise L, Watts CH Intimate partner violence and women’s physical and mental health in the WHO multi-country study on women’s health and domestic violence: An observational study. Lancet. 2008;371(9619):1165–1172. 10.1016/S0140-6736(08)60522-X 18395577

[44] Pico-Alfonso MA, Garcia-Linares MI, Celda-Navarro N, Blasco-Ros C, Echeburúa E, Martinez M The impact of physical, psychological, and sexual intimate male partner violence on women’s mental health: Depressive symptoms, posttraumatic stress disorder, state anxiety, and suicide. J Womens Health. 2006;15(5):599–611. 10.1089/jwh.2006.15.599 16796487

[45] Beck AT, Steer RA, Epstein N, Brown G Psychological assessment. J Consult Clin Psychol. 1990;2(2):191 10.1037/1040-3590.2.2.191

[46] Devries KM, Mak JY, Bacchus LJ, et al Intimate partner violence and incident depressive symptoms and suicide attempts: A systematic review of longitudinal studies. PLoS Med [serial online]. 2013;10(5):e1001439 Available from: http://www.pubmedcentral.nih.gov/articlerender.fcgi?artid=3646718&tool=pmcentrez&rendertype=abstract 10.1371/journal.pmed.1001439 PMC3646718 23671407

[47] Khalifeh H, Dean K Gender and violence against people with severe mental illness. Int Rev Psychiatry. 2010;22(5):535–546. 10.3109/09540261.2010.506185 21047165

[48] Hegarty K Prevalence and associations of partner abuse in women attending general practice: A cross-sectional survey. Aust N Z J Public Health. 2002;26(5):437–442. 10.1111/j.1467-842X.2002.tb00344.x 12413288

